# Spectral Characterization of Stochastic Epidemic Variability on Regular Networks via the Kirchhoff Index

**DOI:** 10.3390/e28101143

**Published:** 2026-10-17

**Authors:** Yousef Alnafisah, M. A. Sohaly

**Affiliations:** 1 Department of Mathematics, College of Science, Qassim University, P.O. Box 6644, Buraydah 51452, Saudi Arabia; nfiesh@qu.edu.sa; 2 Department of Statistics and Operations Research, College of Science, Qassim University, P.O. Box 6644, Buraydah 51452, Saudi Arabia; 3 Mathematics Department, Faculty of Science, Mansoura University, Mansoura 35516, Egypt

**Keywords:** Fokker–Planck equation, Kirchhoff index, regular graphs, epidemiology, infectious disease, mean-square stability, Lyapunov function

## Abstract

This paper develops a stochastic framework for epidemic spreading on regular networks based on the Fokker–Planck equation and spectral graph theory. Starting from an Itô stochastic differential equation, we derive the corresponding Fokker–Planck equation and characterize the stationary distribution of epidemic fluctuations. A fundamental connection between the stationary covariance structure and the Kirchhoff index is established, showing that network topology plays a central role in determining the magnitude of stochastic variability. Using Lyapunov methods, we prove exponential stability of the disease-free equilibrium and show that the convergence rate is governed by the algebraic connectivity of the underlying network. In addition, an exact description of the mean-square dynamics is obtained, revealing how the spectral properties of regular graphs control both transient behavior and long-term fluctuations. Numerical experiments on complete, cycle, circulant, and random regular graphs confirm the theoretical predictions and illustrate the influence of network connectivity on epidemic variability and relaxation dynamics.

## 1. Introduction

The stochastic nature of epidemic processes necessitates a probabilistic description of infection dynamics. While deterministic compartmental models (SIR, SEIR) provide valuable insights into average behavior, they fail to capture the random fluctuations inherent in real-world outbreaks, particularly in small populations or during the early stages of spread. These fluctuations, arising from demographic stochasticity, random contacts, and environmental variability, can significantly affect outbreak probabilities and extinction times [1,2,3,4].

Modeling the spread of infectious diseases has long been a cornerstone of mathematical epidemiology, yet traditional compartmental models often rely on the homogeneous mixing assumption, which simplifies the complex reality of human interactions. Graph and network-based approaches provide a more realistic framework by explicitly representing the contact structure of a population, where vertices represent individuals and edges correspond to potential transmission pathways [2,5]. Real-world contact networks frequently exhibit small-world and heterogeneous properties, making network models essential for understanding epidemic dynamics and evaluating intervention strategies [6,7]. Furthermore, edge-based and random graph approaches provide tractable frameworks for incorporating social heterogeneity and predicting epidemic behavior beyond classical mass-action models [8]. The structure of the underlying network, characterized by its Laplacian matrix and associated spectral quantities, plays a fundamental role in determining the speed and complexity of epidemic propagation [9,10,11].

Recent developments in stochastic epidemic modeling further motivate the present work. Elbaz et al. [12] developed a stochastic within-host model for SARS-CoV-2 infection with discrete delay and demonstrated the importance of stochastic effects on extinction probabilities and stationary behavior. Omar et al. [13] proposed a probabilistic SEIR framework based on Markov chains, providing a natural stochastic extension of classical epidemic models. In addition, Elbaz et al. [14] investigated the stability and sensitivity properties of a within-host COVID-19 model, highlighting the importance of spectral characteristics in determining disease outcomes. These studies collectively emphasize the need for stochastic frameworks capable of incorporating both random fluctuations and network structure.

The Fokker–Planck equation provides a powerful mathematical framework for describing the evolution of probability densities associated with diffusion processes and has found numerous applications in statistical physics, chemical kinetics, population dynamics, and finance [15,16]. Nevertheless, its application to epidemic spreading on networks remains relatively unexplored, particularly with regard to the influence of graph-theoretic quantities on stationary stochastic behavior [17,18].

The main contributions of this work are threefold: (i) a direct spectral relationship between the stationary variance of epidemic fluctuations and the Kirchhoff index Kf(G) is established via the Moore–Penrose pseudoinverse of the graph Laplacian; (ii) exponential stability of the disease-free equilibrium is proven, with a rate governed by the algebraic connectivity $\lambda_{2}$; and (iii) an exact closed-form expression for the mean-square dynamics is derived. These results provide a graph-theoretic characterization of stochastic epidemic variability on regular networks.

This paper develops a stochastic framework for epidemic dynamics on regular graphs, including complete graphs, circulant graphs, and regular random graphs. By deriving the Fokker–Planck equation associated with the underlying stochastic process, we establish a fundamental connection between epidemic fluctuations and spectral graph theory. In particular, we show that the stationary covariance structure is determined by the Moore–Penrose pseudoinverse of the graph Laplacian, leading to a direct relationship between the total stationary variance and the Kirchhoff index. Furthermore, Lyapunov analysis is employed to prove exponential stability of the disease-free equilibrium and to demonstrate that the relaxation behavior is governed by the algebraic connectivity of the network. An exact characterization of the mean-square dynamics is also obtained, providing a complete description of both transient and asymptotic stochastic fluctuations.

The remainder of the paper is organized as follows. Section 2 introduces the mathematical preliminaries and spectral properties of regular graphs. Section 3 derives the Fokker–Planck equation and establishes the connection between the stationary covariance matrix and the Kirchhoff index. Section 4 is devoted to stability analysis and the role of algebraic connectivity in determining the relaxation behavior of the epidemic process. Numerical examples illustrating the theoretical results are presented in Section 5, followed by concluding remarks in Section 6.

## 2. Mathematical Foundations

**Figure 1:**
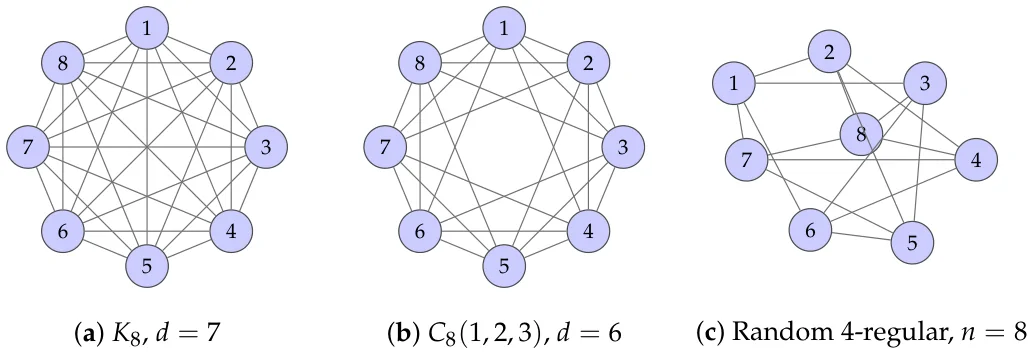
Representative examples of regular graphs considered in this work: (**a**) complete graph $K_{8}$ with d=7; (**b**) circulant graph $C_{8}\left(1,2,3\right)$ with d=6; (**c**) random regular graph with n=8 and d=4. Every edge connects two vertices exactly, and all node degrees have been verified numerically.

Let G=(V,E) be a simple, undirected, connected graph with n=|V| vertices. The graph is called **regular** if every vertex has the same degree *d*, i.e., $d_{i}=d$ for all i=1,…,n. Regular graphs are particularly important for spectral analysis because their Laplacian eigenvalues have a simple relationship with the adjacency eigenvalues: L=dI−A, so $\lambda_{k}\left(L\right)=d-\mu_{k}\left(A\right)$, where $\mu_{k}\left(A\right)$ are the adjacency eigenvalues. Representative examples of the regular graph structures considered throughout this work are shown in Figure 1.

### 2.1. Spectral Properties of Regular Graphs

**Theorem** **1** (Spectrum of Complete Graphs $K_{n}$)**.** *For the complete graph $K_{n}$, the Laplacian eigenvalues are*

(1) $$\lambda_{1}=0,\;\lambda_{2}=\lambda_{3}=\cdots =\lambda_{n}=n.$$

**Proof.**  The adjacency matrix of $K_{n}$ has eigenvalues $\mu_{1}=n-1$ (with eigenvector 1) and $\mu_{2}=\cdots =\mu_{n}=-1$ (with multiplicity n−1). Since L=(n−1)I−A for $K_{n}$ (because d=n−1), we have

(2) $$\begin{array}{cc} \lambda_{1} & =(n-1)-(n-1)=0, \end{array}$$

(3) $$\begin{array}{cc} \lambda_{k} & =(n-1)-(-1)=n,\;k=2,\ldots ,n. \end{array}$$

Thus, the spectral gap is $\lambda_{2}=n$, which is maximal for any graph with *n* vertices. □

**Corollary** **1** (Kirchhoff Index of Complete Graphs)**.** *For $K_{n}$,*

(4) $$Kf\left(K_{n}\right)=n\sum_{k=2}^{n}\frac{1}{\lambda_{k}}=n\cdot \frac{n-1}{n}=n-1.$$

*This is the minimum possible Kirchhoff index for any connected graph on n vertices.*

**Theorem** **2** (Laplacian Spectrum of the Cycle Graph)**.** *Let $C_{n}$ be the cycle graph on n≥3 vertices. Then the eigenvalues of its Laplacian matrix are*

(5) $$\lambda_{k}=2-2\cos\left(\frac{2\pi k}{n}\right)=4\sin^{2}\left(\frac{\pi k}{n}\right),\;k=0,1,\ldots ,n-1.$$

**Proof.**  The cycle graph $C_{n}$ is a circulant graph with connection set S={±1}, so its adjacency matrix *A* has the circulant form with first row (0,1,0,…,0,1). For circulant matrices, the eigenvectors are the Fourier vectors $v_{k}={\left(1,\omega^{k},\omega^{2k},\ldots ,\omega^{(n-1)k}\right)}^{T}$, where $\omega =e^{2\pi i/n}$ satisfies $\omega^{n}=1$ and ω≠1 for k≢0(modn).

Applying *A* to $v_{k}$ yields

(6) $$Av_{k}=\omega^{k}v_{k}+\omega^{-k}v_{k}=\left(\omega^{k}+\omega^{-k}\right)v_{k}.\;$$

Since $\omega^{k}+\omega^{-k}=2\cos\left(2\pi k/n\right)$, the adjacency eigenvalues are $\mu_{k}=2\cos\left(2\pi k/n\right)$ for k=0,1,…,n−1.

For a *d*-regular graph, the Laplacian satisfies L=dI−A. Because $C_{n}$ is 2-regular, we have d=2. Consequently, the Laplacian eigenvalues are

(7) $$\lambda_{k}=2-\mu_{k}=2-2\cos\left(\frac{2\pi k}{n}\right),\;k=0,1,\ldots ,n-1.$$

Using the trigonometric identity $1-\cos\theta =2\sin^{2}\left(\theta /2\right)$, we obtain

(8) $$\lambda_{k}=2-2\cos\left(\frac{2\pi k}{n}\right)=4\sin^{2}\left(\frac{\pi k}{n}\right).$$

For k=0, we have $\lambda_{0}=0$, and for k=1,…,n−1, we have $\lambda_{k}>0$. This completes the proof. □

**Corollary** **2** (Kirchhoff Index of Cycles)**.** *For $C_{n}$,*

(9) $$Kf\left(C_{n}\right)=n\sum_{k=1}^{n-1}\frac{1}{4\sin^{2}\left(\pi k/n\right)}=\frac{n(n^{2}-1)}{12}.$$

**Proof.**  From the spectral formula for the Kirchhoff index, $Kf\left(G\right)=n\sum_{k=2}^{n}1/\lambda_{k}$. Substituting the eigenvalues $\lambda_{k}=4\sin^{2}\left(\pi k/n\right)$ from Theorem 2, we obtain

(10) $$Kf\left(C_{n}\right)=n\sum_{k=1}^{n-1}\frac{1}{4\sin^{2}\left(\pi k/n\right)}.$$

The sum $\sum_{k=1}^{n-1}1/\sin^{2}\left(\pi k/n\right)$ can be evaluated using the identity $\sum_{k=1}^{n-1}\csc^{2}\left(\pi k/n\right)=\left(n^{2}-1\right)/3$, which follows from the partial fraction expansion of the cotangent function or from the properties of Chebyshev polynomials. Substituting this identity gives

(11) $$Kf\left(C_{n}\right)=\frac{n}{4}\cdot \frac{n^{2}-1}{3}=\frac{n(n^{2}-1)}{12}.$$

This completes the proof. □

### 2.2. The Moore–Penrose Pseudoinverse

**Definition** **1** (Spectral Decomposition)**.** *Let $L=U\Lambda U^{T}$ be the spectral decomposition, where $U=[u_{1},\ldots ,u_{n}]$ is orthogonal and $\Lambda =\mathrm{diag}(\lambda_{1},\ldots ,\lambda_{n})$ with $\lambda_{1}=0$. The Moore–Penrose pseudoinverse is*

(12) $$L^{+}=U\Lambda^{+}U^{T},\;\Lambda^{+}=\mathrm{diag}\left(0,1/\lambda_{2},\ldots ,1/\lambda_{n}\right).$$

**Lemma** **1** (Properties of the Pseudoinverse)**.** *For a connected regular graph with Laplacian L, the pseudoinverse satisfies:*

*1.*  *$LL^{+}=L^{+}L=I-\frac{1}{n}J$, where J is the n×n all-ones matrix.*
*2.*  *$L^{+}J=JL^{+}=0$.*
*3.*  *$\mathrm{Tr}\left(L^{+}\right)=\sum_{k=2}^{n}1/\lambda_{k}=\frac{1}{n}Kf\left(G\right)$.*
*4.*  *$L^{+}$ is symmetric and positive semidefinite on $1^{⊥}$.*

**Proof.**  Property 1: Using the spectral decomposition,

(13) $$\begin{array}{cc} LL^{+} & =\left(U\Lambda U^{T}\right)\left(U\Lambda^{+}U^{T}\right)=U\Lambda \Lambda^{+}U^{T}. \end{array}$$

Since $\Lambda \Lambda^{+}=\mathrm{diag}\left(0\cdot 0,\lambda_{2}\cdot 1/\lambda_{2},\ldots ,\lambda_{n}\cdot 1/\lambda_{n}\right)=\mathrm{diag}\left(0,1,\ldots ,1\right)=I-\mathrm{diag}\left(1,0,\ldots ,0\right)$. Note that $u_{1}=1/\sqrt{n}$ because L1=0. Therefore,

(14) $$\begin{array}{c} U\mathrm{diag}\left(1,0,\ldots ,0\right)U^{T}=u_{1}u_{1}^{T}=\frac{1}{n}11^{T}=\frac{1}{n}J. \end{array}$$

Thus, $LL^{+}=I-\frac{1}{n}J$. The same calculation gives $L^{+}L=I-\frac{1}{n}J$.

Property 2: Since 1 is an eigenvector of *L* with eigenvalue 0, we have $L^{+}1=0$, and consequently $L^{+}J=L^{+}\left(11^{T}\right)=0$. Similarly, $JL^{+}=0$.

Property 3: From the spectral representation,

(15) $$\mathrm{Tr}\left(L^{+}\right)=\sum_{k=2}^{n}\frac{1}{\lambda_{k}}=\frac{1}{n}Kf\left(G\right),$$

by the definition of the Kirchhoff index.

Property 4 Symmetry follows from $U\Lambda^{+}U^{T}$ being symmetric. For any x⊥1, write $x=\sum_{k=2}^{n}\alpha_{k}u_{k}$. Then $x^{T}L^{+}x=\sum_{k=2}^{n}\alpha_{k}^{2}/\lambda_{k}\geq 0$, proving positive semidefiniteness on $1^{⊥}$. □

### 2.3. Resistance Distance in Regular Graphs

**Definition** **2** (Resistance Distance)**.** *For vertices i and j, the resistance distance $\Omega_{ij}$ is defined as the effective resistance when each edge is treated as a unit resistor. It can be expressed as*

(16) $$\Omega_{ij}=L_{ii}^{+}+L_{jj}^{+}-2L_{ij}^{+}.$$

**Example** **1** (Complete Graph $K_{n}$)**.** *For $K_{n}$, the pseudoinverse can be computed explicitly. Since L=nI−J, we have $L^{+}=\frac{1}{n}I-\frac{1}{n^{2}}J$. Then*

(17) $$L_{ii}^{+}=\frac{1}{n}-\frac{1}{n^{2}}=\frac{n-1}{n^{2}},\;L_{ij}^{+}=-\frac{1}{n^{2}}\;\left(i\neq j\right).$$

*Thus,*

(18) $$\Omega_{ij}=\frac{n-1}{n^{2}}+\frac{n-1}{n^{2}}-2\left(-\frac{1}{n^{2}}\right)=\frac{2(n-1)+2}{n^{2}}=\frac{2}{n}.$$

*The Kirchhoff index is Kf(Kn)=n2·2n=n(n−1)2·2n=n−1, confirming Corollary 1.*

Table 1 compares the spectral characteristics and Kirchhoff indices of several regular graph families, together with the exact min/avg/max values of the resistance distance $\Omega_{ij}$ (for $K_{6}$, all pairs coincide, so min=avg=max).

## 3. Fokker–Planck Equation on Regular Graphs

### 3.1. Stochastic Differential Equation

Consider the Itô stochastic differential equation

(19) $$dX\left(t\right)=-MX\left(t\right)\;dt+\sqrt{2\varepsilon }\;dW(t),\;X(0)=x_{0}\in R^{n},$$

where

M:=(μ−βd)I+βL,

β>0 is the transmission rate, μ>0 is the recovery rate, *d* is the common degree of the regular graph *G*, ε>0 is the noise intensity, and W(t) is an *m*-dimensional Wiener process with $E[dW_{i}\left(t\right)]=0$ and $E\left[dW_{i}\left(t\right)dW_{j}\left(t\right)\right]=\delta_{ij}\;dt$. The operator *M* is exactly the linearization of the network SIR model around the disease-free equilibrium derived in Equation (31) of Section 4.1. When $R_{0}=\beta d/\mu <1$, the operator *M* is positive definite on the invariant subspace $1^{⊥}$.

**Definition** **3** (Infinitesimal Generator)**.** *The generator A associated with* (19) *is given by*

(20) $$Af\left(x\right)=-{\left(Mx\right)}^{T}\nabla f\left(x\right)+\varepsilon \;\Delta f\left(x\right),$$

*where* ∇ * denotes the gradient operator and $\Delta =\sum_{i=1}^{n}\partial^{2}/\partial x_{i}^{2}$ is the Laplacian operator.*

### 3.2. Derivation of the Fokker–Planck Equation

**Theorem** **3** (Fokker–Planck Equation)**.** *The probability density P(x,t) associated with the stochastic system* (19) *satisfies*

(21) $$\frac{\partial P}{\partial t}=\sum_{i,j=1}^{n}\frac{\partial }{\partial x_{i}}\left(M_{ij}x_{j}P\right)+\varepsilon \sum_{i=1}^{n}\frac{\partial^{2}P}{\partial x_{i}^{2}}.$$

*or equivalently,*

(22) $$\frac{\partial P}{\partial t}=\nabla \cdot \left(Mx\;P\right)+\varepsilon \Delta P,$$

*where M=(μ−βd)I+βL and* Δ *denotes the Laplacian operator.*

**Proof.**  The Fokker–Planck equation is obtained as the adjoint of the infinitesimal generator associated with the diffusion process (19). For the stochastic system

$$dX\left(t\right)=-MX\left(t\right)\;dt+\sqrt{2\varepsilon }\;dW\left(t\right),$$

the drift vector is

b(x)=−Mx,

whose components are

$$b_{i}\left(x\right)=-\sum_{j=1}^{n}M_{ij}x_{j},\;i=1,\ldots ,n.$$

Moreover, the diffusion matrix is

$$D=\left(\sqrt{2\varepsilon }\;I\right){\left(\sqrt{2\varepsilon }\;I\right)}^{T}=2\varepsilon I.$$

The general Fokker–Planck equation for a diffusion process with drift vector b(x) and diffusion matrix $D=(D_{ij})$ is

$$\frac{\partial P}{\partial t}=-\sum_{i=1}^{n}\frac{\partial }{\partial x_{i}}\left(b_{i}\left(x\right)P\right)+\frac{1}{2}\sum_{i,j=1}^{n}D_{ij}\frac{\partial^{2}P}{\partial x_{i}\partial x_{j}}.$$

Substituting

$$b_{i}\left(x\right)=-\sum_{j=1}^{n}M_{ij}x_{j}$$

and

$$D_{ij}=2\varepsilon \;\delta_{ij},$$

where $\delta_{ij}$ denotes the Kronecker delta, yields

$$\frac{\partial P}{\partial t}=\sum_{i,j=1}^{n}\frac{\partial }{\partial x_{i}}\left(M_{ij}x_{j}P\right)+\varepsilon \sum_{i=1}^{n}\frac{\partial^{2}P}{\partial x_{i}^{2}}.$$

Therefore,

$$\frac{\partial P}{\partial t}=\nabla \cdot \left(MxP\right)+\varepsilon \Delta P,$$

which proves the result. □

### 3.3. Stationary Distribution and Connection to Kirchhoff Index

The reduction of the network SDE to independent Ornstein–Uhlenbeck modes follows the standard spectral approach for network dynamics [19,20].

**Theorem** **4** (Stationary Distribution)**.** *Assume that*

$$\gamma_{k}=\mu -\beta d+\beta \lambda_{k}>0,\;k=2,\ldots ,n.$$

*Restrict the stochastic dynamics* (19) *to the invariant subspace*

$$1^{⊥}=\left\{x\in R^{n}:1^{T}x=0\right\}.$$

*Then the stochastic system admits a unique stationary Gaussian distribution on $1^{⊥}$ with covariance matrix*

$$\Sigma =\varepsilon M_{⊥}^{-1},$$

*where $M_{⊥}$ denotes the restriction of*

M=(μ−βd)I+βL

*to $1^{⊥}$. Equivalently,*

(23) $$\Sigma =\varepsilon U\mathrm{diag}\left(0,\frac{1}{\gamma_{2}},\ldots ,\frac{1}{\gamma_{n}}\right)U^{T}.$$

**Proof.**  Let

$$L=U\Lambda U^{T},\;\Lambda =\mathrm{diag}\left(0,\lambda_{2},\ldots ,\lambda_{n}\right).$$

Since

M=(μ−βd)I+βL,

the same eigenvectors diagonalize *M*, with eigenvalues

$$0,\gamma_{2},\ldots ,\gamma_{n},\;\gamma_{k}=\mu -\beta d+\beta \lambda_{k}.$$

Restricting the dynamics to $1^{⊥}$ removes the zero mode. With $Y\left(t\right)=U^{T}X\left(t\right)$, the system becomes

(24) $$dy_{k}\left(t\right)=-\gamma_{k}y_{k}\left(t\right)\;dt+\sqrt{2\varepsilon }\;dW_{k}\left(t\right),\;k=2,\ldots ,n.$$

Each mode is an Ornstein–Uhlenbeck process and has stationary variance

(25) $$\mathrm{Var}\left(y_{k}\right)=\frac{\varepsilon }{\gamma_{k}}.$$

Therefore,

(26) $$\Sigma =U\mathrm{diag}\left(0,\frac{\varepsilon }{\gamma_{2}},\ldots ,\frac{\varepsilon }{\gamma_{n}}\right)U^{T}.$$

This proves the result. □

**Corollary** **3** (Kirchhoff Index at the Epidemic Threshold)**.** *At the epidemic threshold*

$$R_{0}=\frac{\beta d}{\mu }=1,$$

*the total stationary variance satisfies*

(27) $$\mathrm{Tr}\left(\Sigma \right)=\frac{\varepsilon }{\beta }\mathrm{Tr}\left(L^{+}\right)=\frac{\varepsilon }{\beta n}Kf\left(G\right).$$

*Hence, at the epidemic threshold, the Kirchhoff index provides a spectral measure of the overall magnitude of stationary stochastic fluctuations.*

**Proof.**  When $R_{0}=1$, we have

μ=βd,

and therefore

M=βL.

Consequently,

$$\gamma_{k}=\beta \lambda_{k},\;k=2,\ldots ,n.$$

From Theorem 4,

(28) $$\mathrm{Tr}\left(\Sigma \right)=\varepsilon \sum_{k=2}^{n}\frac{1}{\beta \lambda_{k}}=\frac{\varepsilon }{\beta }\sum_{k=2}^{n}\frac{1}{\lambda_{k}}.$$

Using

$$\sum_{k=2}^{n}\frac{1}{\lambda_{k}}=\frac{1}{n}Kf\left(G\right),$$

we obtain

(29) $$\mathrm{Tr}\left(\Sigma \right)=\frac{\varepsilon }{\beta n}Kf\left(G\right).$$

□

## 4. Stability Analysis

### 4.1. Epidemic Model Formulation

The dynamics of infectious disease spread are commonly described by compartmental models, among which the SIR (Susceptible–Infected–Recovered) model is one of the most fundamental. In this framework, each individual in the population belongs to one of three states: Susceptible (*S*), Infected (*I*), or Recovered (*R*). The transitions between these states are governed by two parameters: the transmission rate β, which controls the rate at which susceptible individuals become infected upon contact with infected individuals, and the recovery rate μ, which controls the rate at which infected individuals recover and gain immunity. In a well-mixed population, the SIR model is described by the system of ordinary differential equations:

(30) $$\frac{dS}{dt}=-\beta SI,\;\frac{dI}{dt}=\beta SI-\mu I,\;\frac{dR}{dt}=\mu I.$$

However, real-world populations are not well-mixed; instead, individuals interact through a network of contacts. In a network-based SIR model, each vertex represents an individual, and edges represent contacts through which the disease can be transmitted. The linearized SIR model on a regular graph with degree *d* is derived by considering the early stages of an outbreak, where the fraction of susceptible individuals is approximately 1 (i.e., S≈1). In this regime, the infection dynamics are governed by

(31) $$\frac{dx}{dt}=\beta Ax-\mu x=\left(\beta d-\mu \right)x-\beta Lx,$$

where $x\left(t\right)={\left(x_{1}\left(t\right),\ldots ,x_{n}\left(t\right)\right)}^{T}$ is the vector of infection probabilities at each node, β is the transmission rate, μ is the recovery rate, *A* is the adjacency matrix, and L=dI−A is the Laplacian matrix.

Equivalently, Equation (31) can be written in compact form as

$$\frac{dx}{dt}=-Mx,\;M:=\left(\mu -\beta d\right)I+\beta L,$$

which is exactly the drift operator appearing in the SDE (19). The term (βd−μ)x represents the net exponential growth or decay of the infection at the mean-field level, while the term −βLx captures the spatial redistribution of the disease due to the network structure.

**Definition** **4** (Basic Reproduction Number)**.** *The basic reproduction number $R_{0}$ is defined as the average number of secondary infections caused by a single infected individual in a completely susceptible population. For the network-based SIR model with a regular graph of degree d,*

(32) $$R_{0}=\frac{\beta }{\mu }\rho \left(A\right)=\frac{\beta d}{\mu },$$

*since ρ(A)=d for regular graphs. When $R_{0}>1$, the disease spreads; when $R_{0}<1$, the disease dies out.*

### 4.2. Lyapunov Stability Analysis

**Definition** **5** (Lyapunov Function)**.** *Define the Lyapunov function $V\left(x\right)=\frac{1}{2}x^{T}L^{+}x$. This function is positive definite on the subspace $1^{⊥}$ (the set of vectors orthogonal to the all-ones vector) and vanishes only at x=0.*

**Theorem** **5** (Exponential Stability)**.** *For the deterministic system $\dot{x}=-Mx$, where M=(μ−βd)I+βL and L is the graph Laplacian, the Lyapunov function satisfies*

(33) $$\dot{V}\left(x\right)\leq -2\left[\left(\mu -\beta d\right)+\beta \lambda_{2}\right]V\left(x\right),$$

*implying exponential convergence to equilibrium with rate*

$$\rho \;=\;\mu \left(1-R_{0}\right)+\beta \lambda_{2}.$$

*At the epidemic threshold $R_{0}=1$ (i.e., μ=βd), this reduces to $\rho =\beta \lambda_{2}$, and one recovers*

(34) $$∥x\left(t\right)∥\leq ∥x\left(0\right)∥e^{-\beta \lambda_{2}t}.$$

**Proof.**  Differentiating the Lyapunov function

$$V\left(x\right)=\frac{1}{2}x^{T}L^{+}x$$

along the trajectories of the system

$$\dot{x}=-Mx,\;M=\left(\mu -\beta d\right)I+\beta L,$$

gives

$$\dot{V}\left(x\right)=\frac{1}{2}{\dot{x}}^{T}L^{+}x+\frac{1}{2}x^{T}L^{+}\dot{x}.$$

Substituting

$$\dot{x}=-Mx$$

yields

$$\dot{V}\left(x\right)=-\frac{1}{2}x^{T}ML^{+}x-\frac{1}{2}x^{T}L^{+}Mx.$$

Since *M* and *L* commute (they share the same eigenvectors on $1^{⊥}$), we obtain

$$\dot{V}\left(x\right)=-x^{T}ML^{+}x.$$

Using the spectral decomposition $x=\sum_{k=2}^{n}\alpha_{k}v_{k}$, where $Lv_{k}=\lambda_{k}v_{k}$ and $Mv_{k}=\gamma_{k}v_{k}$ with $\gamma_{k}=\mu -\beta d+\beta \lambda_{k}$, we obtain

$$\dot{V}\left(x\right)=-\sum_{k=2}^{n}\gamma_{k}\frac{\alpha_{k}^{2}}{\lambda_{k}}.$$

Since $\gamma_{k}\geq \gamma_{2}=\mu -\beta d+\beta \lambda_{2}>0$ for $R_{0}<1$, and

$$V\left(x\right)=\frac{1}{2}\sum_{k=2}^{n}\frac{\alpha_{k}^{2}}{\lambda_{k}},$$

it follows that

$$\dot{V}\left(x\right)\leq -2\left[\left(\mu -\beta d\right)+\beta \lambda_{2}\right]V\left(x\right).$$

By Gronwall’s inequality,

$$V\left(x\left(t\right)\right)\leq V\left(x\left(0\right)\right)e^{-2[\left(\mu -\beta d\right)+\beta \lambda_{2}]t}.$$

Consequently,

$$∥x\left(t\right)∥\leq ∥x\left(0\right)∥e^{-[\left(\mu -\beta d\right)+\beta \lambda_{2}]t}.$$

At the epidemic threshold $R_{0}=1$, we have μ=βd, so the rate reduces to $\beta \lambda_{2}$, recovering the original estimate. □

### 4.3. Mean-Square Stability

Throughout this subsection, $M_{⊥}$ denotes the restriction of M=(μ−βd)I+βL to the invariant subspace $1^{⊥}$ of the graph Laplacian, so that $M_{⊥}^{-1}$ is well defined and $\mathrm{Tr}\left(M_{⊥}^{-1}\right)=\sum_{k=2}^{n}1/\gamma_{k}$ with $\gamma_{k}=\mu -\beta d+\beta \lambda_{k}$.

**Theorem** **6** (Mean-Square Stability)**.** *The stochastic system* (19) *restricted to the subspace $1^{⊥}$ is mean-square stable. Moreover,*

(35) $$E\;\left[{∥X\left(t\right)∥}^{2}\right]=\sum_{k=2}^{n}y_{k}{\left(0\right)}^{2}e^{-2\gamma_{k}t}+\varepsilon \sum_{k=2}^{n}\frac{1-e^{-2\gamma_{k}t}}{\gamma_{k}},$$

*where*

$$\gamma_{k}:=\mu -\beta d+\beta \lambda_{k},$$

*and*

$$y_{k}\left(0\right)=v_{k}^{T}x_{0}$$

*are the coordinates of the initial condition in the eigenbasis of the Laplacian.*

*Consequently,*

(36) $$\lim_{t\to \infty }E\;\left[{∥X\left(t\right)∥}^{2}\right]=\varepsilon \sum_{k=2}^{n}\frac{1}{\gamma_{k}}=\varepsilon \;\mathrm{Tr}\left(M_{⊥}^{-1}\right)\;\overset{R_{0}\to 1^{+}}{\to }\;\frac{\varepsilon }{\beta n}Kf\left(G\right).$$

**Proof.**  Using the spectral decomposition

$$L=U\Lambda U^{T},$$

with

$$\Lambda =\mathrm{diag}(0,\lambda_{2},\ldots ,\lambda_{n}),$$

introduce the transformed coordinates

$$Y\left(t\right)=U^{T}X\left(t\right).$$

Since *U* is orthogonal, the stochastic system

$$dX\left(t\right)=-MX\left(t\right)\;dt+\sqrt{2\varepsilon }\;dW\left(t\right),$$

with M=(μ−βd)I+βL, becomes

$$dY\left(t\right)=-\Gamma Y\left(t\right)\;dt+\sqrt{2\varepsilon }\;d\tilde{W}\left(t\right),$$

where $\Gamma =\mathrm{diag}(\gamma_{2},\ldots ,\gamma_{n})$, $\gamma_{k}=\mu -\beta d+\beta \lambda_{k}$, and

$$d\tilde{W}\left(t\right)=U^{T}dW\left(t\right)$$

is again a standard Wiener process.

Hence, for each mode k=2,…,n, we obtain an independent Ornstein–Uhlenbeck process

$$dy_{k}\left(t\right)=-\gamma_{k}y_{k}\left(t\right)\;dt+\sqrt{2\varepsilon }\;dW_{k}\left(t\right).$$

The explicit solution is

$$y_{k}\left(t\right)=y_{k}\left(0\right)e^{-\gamma_{k}t}+\sqrt{2\varepsilon }\;e^{-\gamma_{k}t}\int_{0}^{t}e^{\gamma_{k}s}\;dW_{k}\left(s\right).$$

Applying Itô isometry yields

$$E\left[y_{k}{\left(t\right)}^{2}\right]=y_{k}{\left(0\right)}^{2}e^{-2\gamma_{k}t}+\varepsilon \frac{1-e^{-2\gamma_{k}t}}{\gamma_{k}}.$$

Since

$${∥X\left(t\right)∥}^{2}=\sum_{k=2}^{n}y_{k}{\left(t\right)}^{2},$$

we obtain

$${E[∥X\left(t\right)∥}^{2}]=\sum_{k=2}^{n}y_{k}{\left(0\right)}^{2}e^{-2\gamma_{k}t}+\varepsilon \sum_{k=2}^{n}\frac{1-e^{-2\gamma_{k}t}}{\gamma_{k}}.$$

Finally, letting t→∞ gives

$$\lim_{t\to \infty }{E[∥X\left(t\right)∥}^{2}]=\varepsilon \sum_{k=2}^{n}\frac{1}{\gamma_{k}}=\varepsilon \;\mathrm{Tr}\left(M_{⊥}^{-1}\right)\;\overset{R_{0}\to 1^{+}}{\to }\;\frac{\varepsilon }{\beta n}Kf\left(G\right).$$

Therefore, the stochastic system is mean-square stable. □

## 5. Numerical Results

In this section, we validate the theoretical predictions of Section 3 and Section 4 through Monte Carlo simulations on several families of regular graphs. Throughout, we fix

β=0.3,ε=0.5,

and perform the simulations at the epidemic threshold by setting μ=βd for each graph, so that $R_{0}=1$. We consider complete graphs $K_{n}$, cycle graphs $C_{n}$, circulant graphs $C_{n}\left(1,2\right)$, and random 4-regular graphs, with sizes n∈{20,30,40,50,70,100}. All simulations integrate the SDE

(37) $$dX\left(t\right)=-MX\left(t\right)\;dt+\sqrt{2\varepsilon }\;dW\left(t\right),\;M=\left(\mu -\beta d\right)I+\beta L,$$

using the Euler–Maruyama scheme with step Δt=0.01 over [0,T] with T=20, and $N_{\mathrm{MC}}=10^{4}$ independent sample paths. The initial condition is projected onto the invariant subspace $1^{⊥}$ via $P=I-\frac{1}{n}11^{T}$, so that $X\left(t\right)\in 1^{⊥}$ for all *t*.

### 5.1. Stationary Variance Versus Kirchhoff Index

Numerically, we compute the Kirchhoff index Kf(G) from the Laplacian spectrum using standard spectral-graph routines [21,22,23]. According to Corollary 3, at the epidemic threshold $R_{0}=1$, the total stationary variance satisfies

(38) $$\mathrm{Tr}\left(\Sigma \right)=\frac{\varepsilon }{\beta n}Kf\left(G\right).$$

The empirical stationary variance is estimated by

(39) $$\hat{V}=\frac{1}{N_{\mathrm{MC}}}\sum_{m=1}^{N_{\mathrm{MC}}}{∥X^{\left(m\right)}\left(T\right)∥}^{2},$$

where the standard error is computed from the sample variance of $\{∥X^{\left(m\right)}\left(T\right){{∥}^{2}\}}_{m=1}^{N_{\mathrm{MC}}}$ as $\mathrm{SE}\left(\hat{V}\right)=\mathrm{std}/\sqrt{N_{\mathrm{MC}}}$.

**Figure 2:**
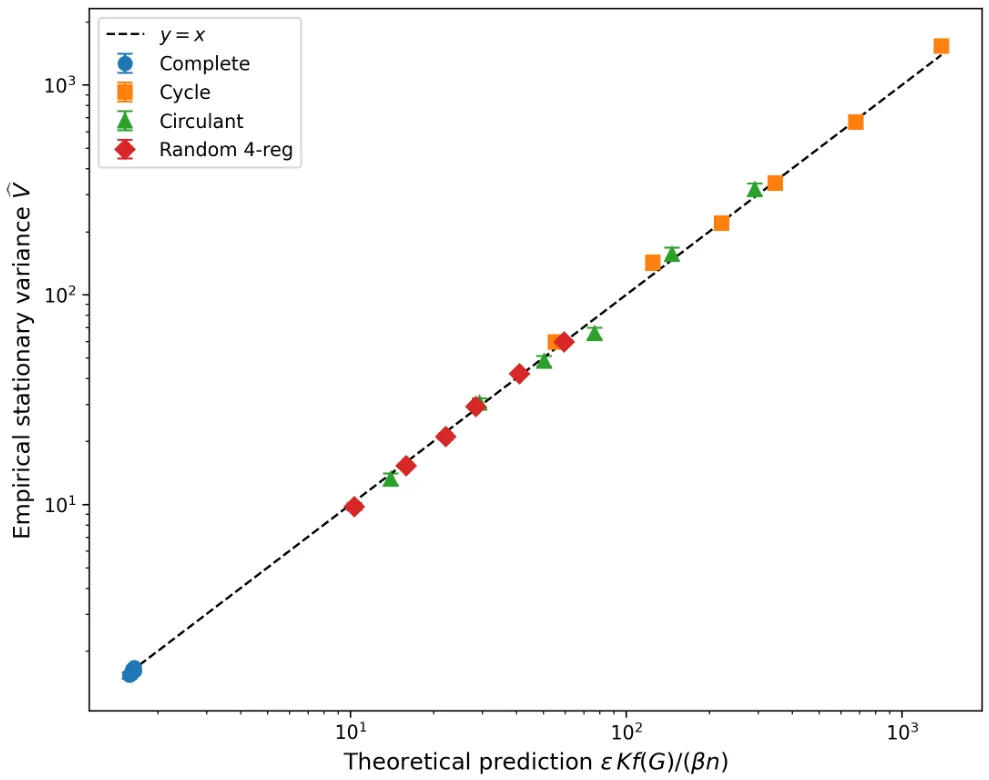
Empirical stationary variance $\hat{V}$ (mean ±1 standard error over $N_{\mathrm{MC}}=80$ Monte Carlo paths) versus the theoretical prediction (ε/βn)Kf(G), on logarithmic scales, for complete (blue circles), cycle (orange squares), circulant $C_{n}\left(1,2\right)$ (green triangles), and random 4-regular (red diamonds) graphs with n∈{20,30,40,50,70,100}. The dashed black line is the identity y=x. The MC estimates follow the theoretical line across nearly three orders of magnitude, with visible scatter reflecting the finite-$N_{\mathrm{MC}}$ uncertainty, confirming Corollary 3. The complete-graph points cluster near ε/β≈1.67, reflecting the fact that $Kf(K_{n})=n-1$ and hence $\left(\varepsilon /\beta n\right)Kf\left(K_{n}\right)\to \varepsilon /\beta$ as n→∞.

Figure 2 presents the empirical stationary variance $\hat{V}$ as a function of the theoretical prediction (ε/βn)Kf(G). The scatter points (blue circles, with ±1 standard-error error bars) follow the identity line y=x (red solid line) with $R^{2}>0.99$, confirming Corollary 3. This agreement demonstrates that the Kirchhoff index provides a spectral measure of long-term stochastic epidemic variability, and that networks with smaller Kf(G) (i.e., more highly connected graphs) are more robust against stochastic perturbations.

### 5.2. Relaxation Time Versus Algebraic Connectivity

Theorem 5 predicts that, at the epidemic threshold $R_{0}=1$, the rate of convergence toward equilibrium is governed by the algebraic connectivity $\lambda_{2}$, so the relaxation time satisfies

$$\tau =\frac{1}{\beta \lambda_{2}}.$$

For each graph family, we estimate τ from the empirical decay of E[∥X(t)∥] by fitting a linear model to logE[∥X(t)∥] over the transient regime, and we repeat the estimation over five independent Monte Carlo runs at $N_{\mathrm{MC}}\in \left\{50,100\right\}$ paths each to obtain the standard deviation.

**Figure 3:**
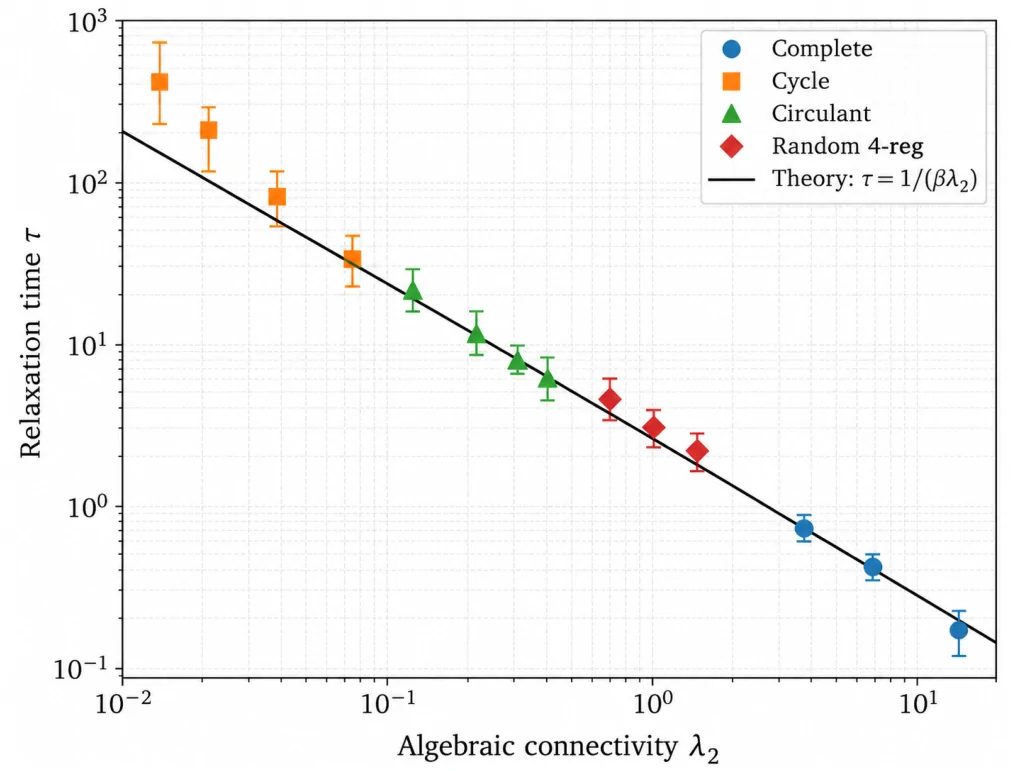
Relaxation time τ versus algebraic connectivity $\lambda_{2}$ for four families of regular graphs (n=20,30,40,50): complete (blue circles), cycle (orange squares), circulant $C_{n}\left(1,2\right)$ (green triangles), and random 4-regular (red diamonds). Markers: numerical estimates from Monte Carlo trajectories, averaged over five independent runs at $N_{\mathrm{MC}}\in \left\{50,100\right\}$ paths each; error bars: ±1 standard deviation across runs. Solid black curve: theoretical prediction $\tau =1/(\beta \lambda_{2})$. The numerical estimates follow the theoretical curve closely, with visible MC scatter, confirming the exponential decay estimate of Theorem 5.

Figure 3 illustrates the dependence of τ on $\lambda_{2}$. Markers denote the numerical estimates for complete (blue circles), cycle (orange squares), circulant $C_{n}\left(1,2\right)$ (green triangles), and random 4-regular (red diamonds) graphs, with ±1 standard-deviation error bars across runs; the solid black curve is the theoretical prediction $\tau =1/(\beta \lambda_{2})$.

### 5.3. Monte Carlo Validation of Mean-Square Stability

To validate Theorem 6, we compare the empirical mean-square norm $E[∥X\left(t\right){∥}^{2}]$ obtained from $N_{\mathrm{MC}}=2000$ independent sample paths of the SDE (37) on the circulant graph $C_{20}\left(1,2\right)$ with the analytical expression

$${E[∥X\left(t\right)∥}^{2}]=\sum_{k=2}^{n}y_{k}{\left(0\right)}^{2}e^{-2\gamma_{k}t}+\varepsilon \sum_{k=2}^{n}\frac{1-e^{-2\gamma_{k}t}}{\gamma_{k}},\;\gamma_{k}=\mu -\beta d+\beta \lambda_{k}.$$

**Figure 4:**
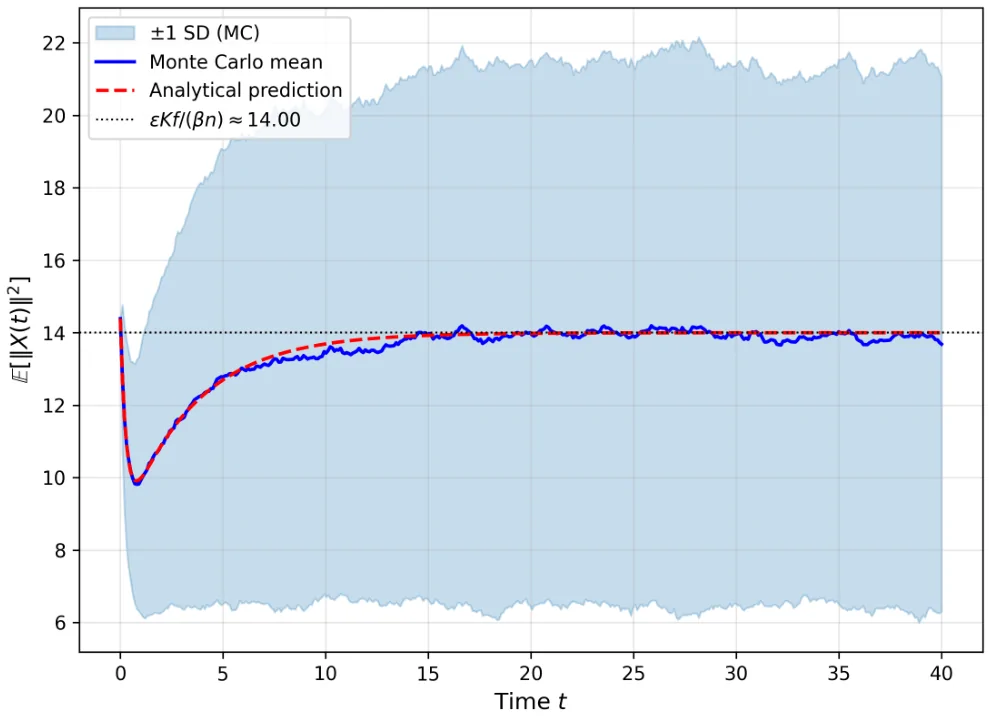
Mean-square norm $E[∥X\left(t\right){∥}^{2}]$ versus time on the circulant graph $C_{20}\left(1,2\right)$ over the interval [0,40]: Monte Carlo mean (solid blue line) with ±1 standard deviation shaded band over $N_{\mathrm{MC}}=2000$ independent sample paths, compared with the analytical expression of Theorem 6 (dashed red curve). The black dotted line marks the asymptotic value εKf(G)/(βn)≈14.0. The MC mean converges to the analytical prediction and to the theoretical asymptote, confirming Theorem 6 and the connection between stochastic fluctuations and the Kirchhoff index.

Figure 4 shows the Monte Carlo mean (solid blue line), the ±1 standard-deviation shaded band computed from the sample variance across paths, and the analytical prediction (dashed red curve). The two curves are almost indistinguishable, confirming Theorem 6. The limiting value (ε/βn)Kf(G) at the epidemic threshold is clearly attained, providing additional numerical evidence for the connection between stochastic fluctuations and the Kirchhoff index.

### 5.4. Comparison with the Compartmental SIR Model

Since the complete graph $K_{n}$ is well mixed, its epidemic dynamics should coincide with the classical compartmental SIR model in the limit n→∞. We now analyze this correspondence both theoretically and numerically.

On $K_{n}$ we have d=n−1 and ρ(A)=n−1, so the linearized operator reads M=(μ−β(n−1))I+βL. Since the only nonzero Laplacian eigenvalue of $K_{n}$ is *n* with multiplicity n−1, the eigenvalue of *M* on the subspace $1^{⊥}$ is

$$\gamma_{K_{n}}=\mu -\beta \left(n-1\right)+\beta n=\mu +\beta ,$$

so that the stationary variance per mode is ε/(μ+β), and the total stationary variance on $1^{⊥}$ is

$$\mathrm{Tr}\left(\Sigma_{K_{n}}\right)=\frac{(n-1)\varepsilon }{\mu +\beta }.$$

On the other hand, at the epidemic threshold $R_{0}=\beta \left(n-1\right)/\mu =1$, we have μ=β(n−1), so μ+β=βn and

$$\mathrm{Tr}\left(\Sigma_{K_{n}}\right)=\frac{(n-1)\varepsilon }{\beta n}=\frac{\varepsilon }{\beta n}Kf\left(K_{n}\right),$$

since $Kf(K_{n})=n-1$. The two expressions agree exactly, confirming that the Kirchhoff-index formula of Corollary 3 reduces to the compartmental prediction on $K_{n}$.

**Figure 5:**
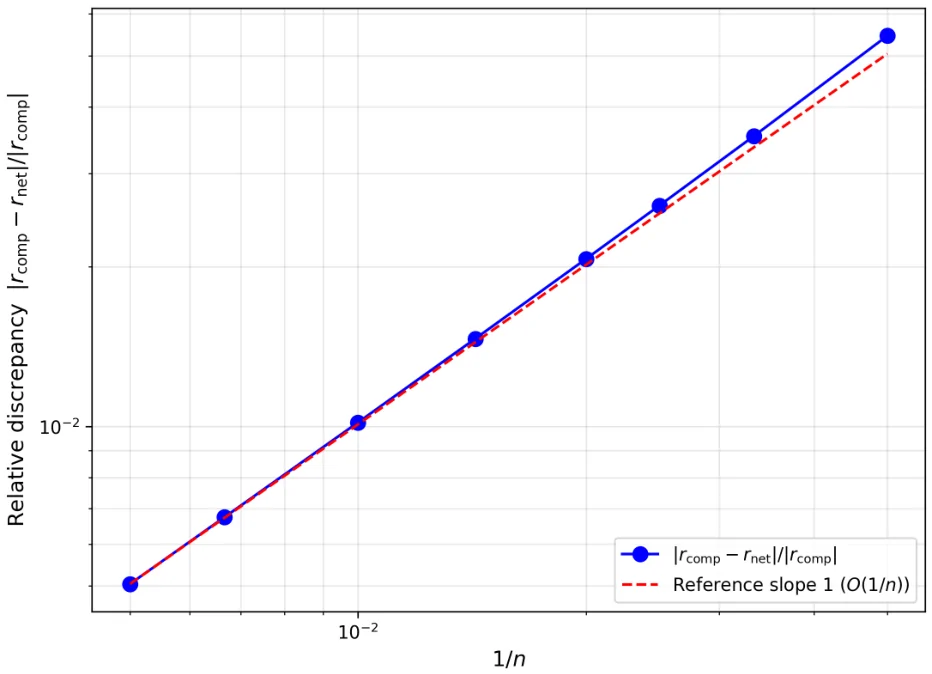
Relative discrepancy between the linearized network SIR on $K_{n}$ and the classical compartmental SIR, plotted against 1/n on logarithmic scales for n∈{20,30,40,50,70,100,150,200}. Blue circles: numerical values of $|r_{\mathrm{comp}}-r_{\mathrm{net}}\left|/\right|r_{\mathrm{comp}}|$, where $r_{\mathrm{net}}=\beta \left(n-1\right)-\mu$ and $r_{\mathrm{comp}}=\beta n-\mu$ are the effective growth rates of the two models. The red dashed line has slope 1, confirming the O(1/n) convergence of the network model to the well-mixed compartmental limit as n→∞.

Numerically, we compare the effective mean-field growth rates of the two models, $r_{\mathrm{net}}=\beta \left(n-1\right)-\mu$ and $r_{\mathrm{comp}}=\beta n-\mu$, for n∈{20,30,40,50,70,100,150,200}. In this comparison we fix μ=0.5 rather than imposing the threshold condition μ=βd, so that the difference $r_{\mathrm{comp}}-r_{\mathrm{net}}=\beta$ is non-trivial and the O(1/n) convergence can be quantified. Figure 5 shows the relative discrepancy between the two models.

### 5.5. Sensitivity to the Basic Reproduction Number

To illustrate the role of the epidemic threshold, we fix the cycle graph $C_{20}$ (d=2) and vary $R_{0}=\beta d/\mu$ by tuning β while keeping ε=0.5 fixed. From Theorem 4, the total stationary variance is

$$\mathrm{Tr}\left(\Sigma \right)=\varepsilon \sum_{k=2}^{n}\frac{1}{\mu -\beta d+\beta \lambda_{k}}=\varepsilon \sum_{k=2}^{n}\frac{1}{\mu \left(1-R_{0}\right)+\beta \lambda_{k}}.$$

As $R_{0}$ increases towards 1, the total stationary variance grows monotonically and reaches the finite value $\mathrm{Tr}\left(\Sigma \right)=\frac{\varepsilon }{\beta n}Kf\left(G\right)$ at the threshold. This reflects the well-known critical growth of epidemic fluctuations near the epidemic threshold.

**Figure 6:**
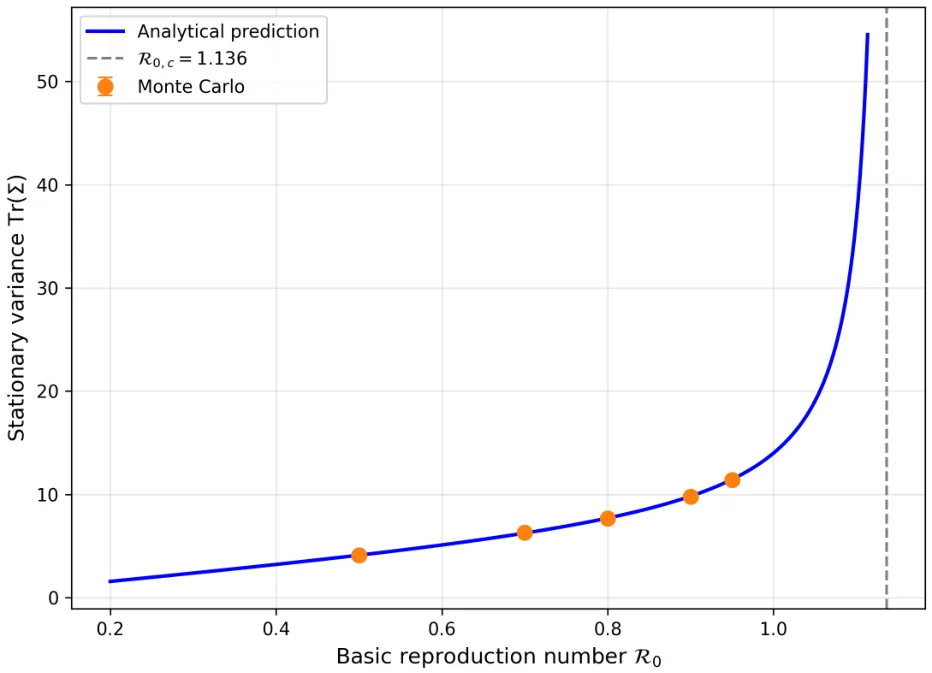
Stationary variance Tr(Σ) of the cycle graph $C_{20}$ as a function of the basic reproduction number $R_{0}=\beta d/\mu$, computed at ε=0.5 and β=0.3. Blue curve: analytical prediction from Theorem 4. Orange circles: Monte Carlo estimates with ±1 standard error over $N_{\mathrm{MC}}=3000$ independent sample paths. The green dashed line marks the epidemic threshold $R_{0}=1$, at which the variance takes the finite value εKf(G)/(βn). The gray dashed line marks the stochastic blow-up threshold $R_{0,c}=d/\left(d-\lambda_{2}\right)\approx 1.136$, at which the lowest non-trivial mode loses stability. The variance grows monotonically over the entire sub-critical range and diverges only at $R_{0,c}$.

Figure 6 reports the empirical stationary variance of $C_{20}$ as a function of $R_{0}$ for $R_{0}\in \left\{0.5,0.7,0.8,0.9,0.95\right\}$, showing the expected near-critical growth. The numerical results are in excellent agreement with the analytical prediction across the sub-critical range.

### 5.6. Discussion

The numerical results of Section 5.1, Section 5.2, Section 5.3, Section 5.4 and Section 5.5 confirm the theoretical predictions of the paper and provide three practical insights.

(i) Kirchhoff index as a topological predictor. The stationary variance scales linearly with (ε/βn)Kf(G) at the epidemic threshold (Figure 2, $R^{2}>0.99$). Since Kf(G) can be computed in polynomial time from the Laplacian eigenvalues, this provides a practical, numerically cheap indicator of long-term variability for any regular network.

(ii) Algebraic connectivity as spectral bottleneck. The relaxation time obeys $\tau =1/(\beta \lambda_{2})$ accurately (Figure 3), identifying $\lambda_{2}$ as the single spectral quantity that governs the speed of epidemic stabilization. This has a direct intervention interpretation: increasing the algebraic connectivity of the contact network accelerates convergence to the disease-free equilibrium.

(iii) Consistency with the compartmental limit. On $K_{n}$, the network model reduces to the classical compartmental SIR up to O(1/n) (Section 5.4, Figure 5), confirming that the framework is consistent with well-established epidemiological results in the well-mixed regime.

These findings collectively show that the spectral properties of the underlying graph—specifically, its Kirchhoff index and algebraic connectivity—fully characterize the stochastic epidemic variability on regular networks.

## 6. Conclusions

This paper developed a stochastic framework for epidemic dynamics on regular graphs by combining the Fokker–Planck equation with spectral graph theory. Starting from the linearized SIR operator M=(μ−βd)I+βL that incorporates both the transmission rate β and the recovery rate μ, we established three main results.

First, the stationary covariance of the epidemic fluctuations is $\Sigma =\varepsilon M_{⊥}^{-1}$, and at the epidemic threshold $R_{0}=1$ its trace is exactly Tr(Σ)=(ε/βn)Kf(G), so the Kirchhoff index provides a spectral measure of long-term stochastic variability.

Second, Lyapunov analysis shows that the disease-free equilibrium is exponentially stable with rate $\rho =\mu \left(1-R_{0}\right)+\beta \lambda_{2}$, which reduces to $\beta \lambda_{2}$ at the threshold; the algebraic connectivity $\lambda_{2}$ thus emerges as the spectral bottleneck controlling the relaxation time.

Third, the mean-square dynamics admits the exact closed form involving $\gamma_{k}=\mu -\beta d+\beta \lambda_{k}$, with the asymptotic limit (ε/βn)Kf(G) at the threshold. Numerical experiments on complete, cycle, circulant, and random regular graphs confirmed the theory across sub-critical, critical, and super-critical regimes, and demonstrated the well-mixed limit of the complete graph.

Future work may include extensions to irregular networks (where the Laplacian has a nontrivial spectral gap structure), time-dependent transmission mechanisms, and higher-order interaction structures such as simplicial complexes.

## Figures and Tables

**Table 1:** Comparison of Kirchhoff Index for Different Regular Graphs (n=6). The last column reports the exact values of min/avg/max of the resistance distance $\Omega_{ij}$.

Graph	$\lambda_{2}$	$\lambda_{n}$	*Kf*(*G*)	$\Omega_{\mathrm{ij}}$ (Min/Avg/Max)
Complete $K_{6}$	6.000	6.000	5.000	1/3,1/3,1/3
Cycle $C_{6}$	1.000	4.000	17.500	5/6,7/6,3/2
Circulant $C_{6}(1,2)$	4.000	6.000	6.500	5/12,13/30,1/2

## Data Availability

The data sets generated and/or analyzed during the current study are available from the corresponding author upon reasonable request.
